# INSTIGO Trial: Evaluation of a Plasma Protein Profile as a Predictive Biomarker for Metastatic Relapse of Triple Negative Breast Cancer

**DOI:** 10.3389/fonc.2021.653370

**Published:** 2021-06-25

**Authors:** Hugo Veyssière, Sejdi Lusho, Ioana Molnar, Myriam Kossai, Maureen Bernadach, Catherine Abrial, Yannick Bidet, Nina Radosevic-Robin, Xavier Durando

**Affiliations:** ^1^ Université Clermont Auvergne, INSERM UMR 1240 « Imagerie Moléculaire et Stratégies Théranostiques », Centre Jean PERRIN, Clermont-Ferrand, France; ^2^ Division de Recherche Clinique, Délégation Recherche Clinique & Innovation, Centre Jean PERRIN, Clermont-Ferrand, France; ^3^ Centre d’Investigation Clinique, UMR501, Clermont-Ferrand, France; ^4^ Département d’anatomie et de cytologie pathologiques, Centre Jean PERRIN, Clermont-Ferrand, France; ^5^ Département d’oncogénétique, Laboratoire d’Oncologie Moléculaire, Centre Jean PERRIN, Clermont-Ferrand, France

**Keywords:** triple negative breast cancer, predictive biomarker, plasma proteins, metastatic relapse, ctDNA, TILs, RNA signature, blood cells

## Abstract

**Background:**

Triple negative breast cancer (TNBC) accounts for 10-20% of breast cancers but has no specific therapy. While TNBC may be more sensitive to chemotherapy than other types of breast cancer, it has a poor prognosis. Most TNBC relapses occur during the five years following treatment, however predictive biomarkers of metastatic relapse are still lacking. High tumour-infiltrating lymphocytes (TILs) levels before and after neo-adjuvant chemotherapy (NAC) are associated with lower relapse risk and longer survival but TILs assessment is highly error-prone and still not introduced into the clinic. Therefore, having reliable biomarker of relapse, but easier to assess, remains essential for TNBC management. Searching for such biomarkers among serum/plasma proteins, circulating tumoral DNA (ctDNA) and blood cells appear relevant.

**Methods:**

This single-centre and prospective study aims to discover predictive biomarkers of TNBC relapse and particularly focuses on plasma proteins. Blood samples will be taken at diagnosis, on the day of first-line or post-NAC surgery, on the day of radiotherapy start, then 6 months and one year after radiotherapy. A blood sample will be taken at the time of metastatic relapse diagnosis. Blood samples will be used for circulating protein quantification, blood cell counts and circulating tumour DNA quantification. A tumour RNA signature, based on the analysis of the RNA expression of 6 genes, will also be tested from the initial biopsy taken routinely. In NAC patients, TILs quantity will be assessed on TNBC pre-treatment biopsy and surgical specimen.

**Ethics and Dissemination:**

INSTIGO belongs to category 2 interventional research on humans. This study has been approved by the SUD*-*EST IV ethics committee and is conducted in accordance with the Declaration of Helsinki and General Data Protection Regulation (GDPR). Study findings will be published in peer-reviewed medical journals.

**Clinical Trial Registration:**

ClinicalTrials.gov, identifier NCT04438681.

## Introduction

Triple negative breast cancer accounts for approximately 10-20% of breast cancers and is characterized by the lack -or by very low - expression of oestrogen and progesterone receptors and the lack of amplification of the gene coding for HER2 (Human Epidermal Growth Factor Receptor 2) (1). TNBC has no specific therapy; chemotherapy, radiotherapy and surgery remain preferred modalities. While TNBC may be more sensitive to chemotherapy than other types of breast cancer, it has a poor prognosis due to its heterogeneity (2–4). Predictive biomarkers of metastatic relapse and type of relapse need to be discovered. Among these predictive biomarkers, it has been shown that a high level of TILs before and after NAC is associated with lower recurrence risk and longer recurrence-free survival (5, 6). However, TIL assessment is error-prone and subject to high inter-evaluator variability despite the existence of standardized recommendations (7, 8). It is also known that a complete pathological response to NAC is associated with a low risk of metastatic relapse in TNBC (9). The determination of biomarkers that are easily quantifiable at diagnosis is essential. The search for predictive biomarkers of metastatic progression among circulating molecules seems relevant (10, 11). It has been shown that high concentrations of proteins involved in inflammation, such as interleukins 6 and 8, or involved in angiogenesis, such as angiopoietin-like protein, are associated with a high risk of metastatic progression of breast cancer (12–14). In TNBC, high blood levels of transforming growth factor-β (TGF-β) and vascular endothelial growth factor-A (VEGF-A) are associated with a high risk of relapse (15). Circulating proteins assays are part of routine clinical testing and have a high sensitivity. Targeted analysis of the blood proteome (serum/plasma), using high throughput techniques such as multiplex ELISA, appears to be an interesting approach for the discovery of new biomarkers. In this context, we propose to conduct a study that measures the concentrations of a set of plasma proteins to evaluate their ability to predict metastatic relapse in patients with TNBC.

The interest in circulating biomarkers also leads us to focus on blood cells which are easily quantifiable and accessible. Blood cells and their ratios [Platelet-to-lymphocyte Ratio (PLR) and Neutrophil-to-lymphocyte Ratio (NLR)] are predictive and prognostic biomarkers of breast cancer (16, 17). In patients with TNBC, a high NLR, reflecting a weak immune response, is associated with a poor response to NAC and a poor survival (18, 19). In addition, plasma from cancer patients contains circulating tumoral DNA (ctDNA) carrying tumour mutations. ctDNA is proving to be another biomarker of interest to study. ctDNA levels make it possible to anticipate the response to treatment and to predict the risk of metastatic relapse (20, 21). They are also a predictive and prognostic biomarker in patients with metastatic breast cancer (22). Combining the characteristics of a plasma protein profile, blood cells levels (PLR, NLR) and ctDNA would provide information on the metastatic potential of a given tumour.

Finally, analyses of tumour RNA expression of a wide range of genes by PAM50 tests, EndoPredict tests, MammaPrint tests or even BluePrint tests allow clinicians to classify breast cancers into molecular subtypes, each corresponding to a specific prognosis and treatment proposal (23–27). However, no such tests exist for TNBCs. The validation of an RNA signature of triple negative breast tumours, established at the Centre Jean Perrin by the analysis of the RNA expression of 6 genes, could provide us an indication on the treatments to be preferred.

## Methods and Study Design

### Study Design

**Figure 1:**
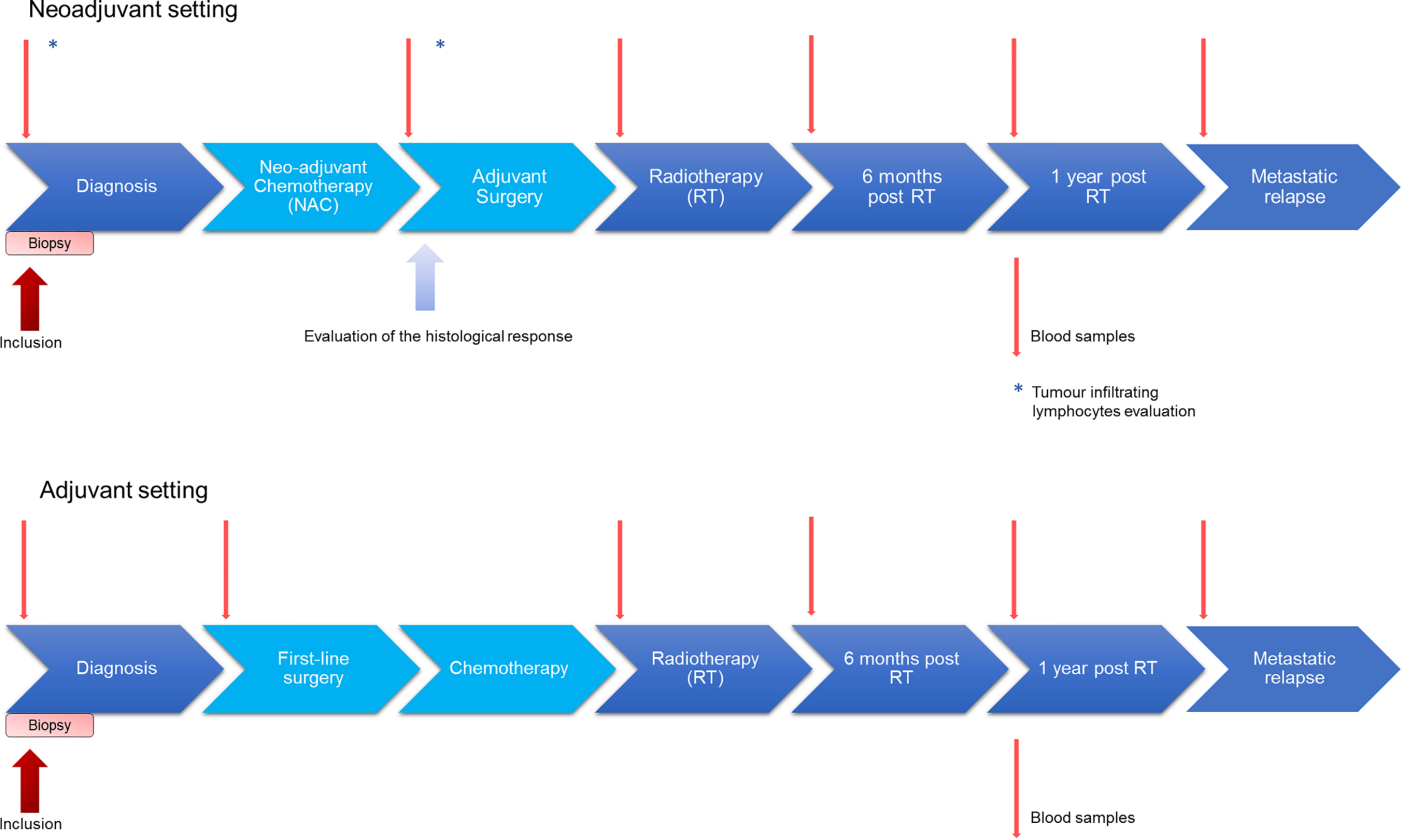
Design of the INSTIGO study. Patients will have blood samples taken at diagnosis, on the day of first-line surgery or post-NAC surgery, on the day of the start of radiotherapy, and 6 months and one year after radiotherapy. If necessary, a blood sample will be taken at the time of diagnosis of metastatic relapse. Blood samples will be used for circulating proteins quantification, blood cells assay and circulating tumour DNA quantification. In neoadjuvant setting, TILs rate will be evaluated from tumour tissue from the biopsy and from the operating room.

This is a single-centre prospective trial designed to evaluate a plasma protein profile as a predictive biomarker for metastatic relapse of TNBC. The estimated duration of patients’ enrolment is 3 years: 90 patients will be enrolled and followed from the patient inclusion until the first metastatic relapse or up to 5 years after the end of treatment if no relapse occurs. Study design is presented in **Figure 1**. TNBC patients will have blood samples taken at diagnosis, on the day of first-line surgery or post-NAC surgery, on the day of radiotherapy start, and 6 months and one year after the end of radiotherapy. Patients receiving neoadjuvant radiation will not be included in the trial. In case of recurrence, a blood sample will be taken at the time of diagnosis of metastatic relapse. Blood samples will be used for the quantification of plasma proteins, for the determination of blood cells and for the quantification of ctDNA. TILs rate evaluation will be performed on tumour tissue from the biopsy and from the operating specimen. The quantification of proteins will be done by multiplex ELISA. The identification of tumoral DNA mutations will be made from extracted DNA from tumour tissue from the operating specimen or from the biopsy. All coding regions, including exons borders and splicing sites, of the ten most frequently muted genes in breast tumours (including TP53, PTEN, PIK3CA, etc) will be captured from sample genomes. Then, targeted regions will be analysed by high throughput sequencing. Targeting several genes will ensure to discover at least one tumour-specific mutation. This mutation will later be quantified in peripheral blood samples. The RNA signature will be generated from transcripts extracted from tumour tissue from the surgical specimen or from the biopsy. A multiplex RT-qPCR will quantify the expression levels of the 6 genes of interest. The expression level of each gene will be multiplied by the corresponding coefficient in order to generate a prognostic score.

### Study Objectives

The primary objective of the INSTIGO trial is to discover a baseline plasma protein profile predictive of metastatic relapse in patients with TNBC (**Table 1**). It also aims to identify a plasma protein profile at different times during patient follow-up that could be predictive of metastatic relapse. Based on a review of the literature INSTIGO focuses on a specific group of proteins: Matrix metalloproteinase 9, tissue inhibitor of metalloproteinase 1, interleukin-6, interleukin-8, interleukin-10, programmed death-ligand 1, stromal cell-derived factor 1, GM-CSF, tyrosine kinase with immunoglobulin and epidermal growth factor-homology domains 2, TGF-β, VEGF-A, hepatocyte growth factor, fibroblast growth factor, CXCL5, CXCL12. Moreover, the INSTIGO study takes interest in others potential biomarkers such as ctDNA, blood cells, TILS, or tumour RNA expression (**Table 1**). Briefly, the objective is to evaluate the ability of those potential biomarkers to predict metastatic relapse in patients with triple-negative breast cancers.

**Table 1:** Primary and secondary objectives.

Primary objective	To discover a baseline plasma protein profile predictive of metastatic relapse in patients with TNBC
Secondary objectives	To discover plasma protein profiles predictive of metastatic relapse in patients with TNBC, assessed at - the day of first surgery or post-CTNA surgery - the day of the radiotherapy start - 6 months and one year after radiotherapy
	To study the relationship between the quantity of tumour infiltrating lymphocytes at diagnosis and metastatic relapse
	To study the relationship between NLR and PLR and metastatic relapse, when those 2 parameters are assessed at - diagnosis - the day of first surgery or post-CTNA surgery
	To study the relationship between plasma levels of circulating tumoral DNA and metastatic relapse - at diagnosis - at the day of first surgery or post-CTNA surgery - at the day of the start radiotherapy start - 6 months and one year after radiotherapy
	To verify prognostic value of a baseline RNA signature
In neoadjuvant setting	To identify a baseline plasma protein profile predictive of histological response to neoadjuvant chemotherapy (NAC)
	To study the relationship between variation in protein concentration, between diagnosis and end of NAC, and histological response to NAC
	To study the relationship between TIL quantity before and after NAC, as well as between the relationship between the baseline TIL level and the level of histological response to NAC
	To study the relationship between the variation of the PLR ​​and the NLR before and after NAC, as well as between the relationship between the baseline PLR and NLR levels and the level of histological response to NAC

### Patient Selection

Inclusion and exclusion criteria are presented in **Table 2**. Briefly, women (18 years or older) with newly diagnosed, histologically proven and never treated primary triple negative breast cancer, and non-metastatic (M0) at diagnosis, will be included.

**Table 2:** Inclusion and non-inclusion criteria.

Inclusion criteria	Female Age ≥ 18 years Newly diagnosed, histologically proven and never treated primary triple negative breast cancer, and non-metastatic (M0) at diagnosis Speaking and understanding French Affiliated to the French Social Security System Able to give informed consent.
Non-inclusion criteria	Patient deprived of liberty by court or administration decision In neoadjuvant situation: neoadjuvant treatment by radiotherapy or hormone therapy Refusal to participate to the study

### Recruitment and Consent

Eligible patients will be offered the opportunity to participate in the study by their oncologist or their surgeon. Patients who agree to participate in this study will provide written informed consent for enrolment. Data obtained will be retained with consent, and any reasons given for withdrawal will be recorded. Participants can withdraw at any time.

### Sample Size Calculation

Given the exploratory nature of the study, and the lack of sufficient data to provide hypotheses in order to perform sample size calculation, the objective is to recruit a maximum of patients. The recruitment capacity for this study is estimated at 90 patients (30 patients per year). An interim analysis at 30 patients will allow us to re-evaluate the recruitment capacity, and to assess the interest of extending the recruitment period, and to re-estimate the number of subjects needed in view of the amount of missing data and the variability of the protein data. A rough estimation allows us to evaluate that with a sample size of 90 patients we would obtain a 95% (Wilson’s) confidence interval for the sensitivity of a 90% predictive score, with a precision of +/- 12%, assuming that the proportion of metastatic relapses at 5 years will be 1/3.

### Data Collections

Data collected are the patient’s age (month and year of birth), pathology, treatments received, response to NAC, clinical and molecular characteristics of the tumour on biopsy and surgical specimen and blood tests at diagnosis, on the day of the first-line surgery, before the start of radiotherapy, 6 months and 1 year after the end of radiotherapy and at the time of metastatic relapse. Data collected will be pseudonymized. Thus, study data will not contain any names or other personal identifiers such as addresses. Patients included in the trial will be identified by a code specific to this trial. The investigator will have access to the correspondence table between the patient’s last name, first name, date of birth and the code assigned in the trial.

### Statistical Analysis

#### Primary Analysis

The predictive plasma profile of metastatic relapse will be investigated using an approach based on the elastic-net method. Firstly, univariate analyses (Wilcoxon-Mann-Whitney tests with *p*-value correction for FDR control) will be performed to evaluate the relationship between the concentration of each protein and the occurrence of relapse. Then, a multivariate logistic regression model with elastic-net regularization will be constructed to allow an intrinsic selection of predictive variables (proteins). If necessary, a selection stabilization algorithm will also be applied. The results of the analysis will therefore be the set of predictive variables selected by the selected model, their regression coefficients, as well as an estimation of the model performance (with associated confidence intervals). However, in the absence of a test dataset the performance of the model can only be estimated by cross-validation.

#### Secondary Analysis

To identify a plasma protein profile that is predictive of metastatic relapse, the same approach as for the main objective will be used.

The relationship between TILs, NLR and PLR levels measured at different times, or ctDNA plasma levels and metastatic relapse will be studied by Wilcoxon-Mann-Whitney tests and logistic regressions. Mixed models will be used to account for repeated data.

The relationship between, on the one hand, the variation in protein concentration, the variation in TILs, or the variation in PLR and NLR levels, between diagnosis and end of NAC, and, on the other hand, the histological response to NAC, will be studied as in the previous point. These aspects are also concerned by an intermediate analysis on the first 30 patients.

An RNA signature predictive of metastatic relapse will be assessed by applying a previously constructed model on the data obtained in the INSTIGO study. The classical indices for the evaluation of a classification (sensitivity, specificity, and precision, among others) will be calculated.

### Trial Status

As of this day 2 patients has been recruited in the INSTIGO trial. Participant recruitment began on 9^th^ November 2020 and is expected to finish in November 2023. The approved protocol is version 15, 04/09/2020.

### Patient and Public Involvement

Neither patients nor the public were involved in the design of this research.

### Ethics and Dissemination

The INSTIGO trial has been approved by an ethics committee (SUD*-*EST IV – Léon Bérard) on September 2020 (ID-RCB number: 2020-A01423-36). It is conducted notably in accordance with the Declaration of Helsinki and General Data Protection Regulation (GDPR). Study data and finding will be published in peer-reviewed medical journals. We plan to present the study and all data at national congresses and conferences.

## Discussion

The discovery of new inexpensive and reliable biomarkers to predict treatment response and metastatic recurrence in TNBC patients remain an important medical need. Such biomarkers would allow oncologists to offer an alternative treatment to TNBC patients with a high risk of metastatic recurrence. During the last years, many proteins appeared to predict clinical behaviour and new biomarkers have been proposed to predict survival and response to chemotherapy in many cases. The INSTIGO trial explores a group of blood proteins expected to be reliable biomarkers. Thus, this study would allow us to determine whether a group of plasma proteins can predict response to neo-adjuvant chemotherapy and metastatic relapse in TNBC. Proteins are easily quantifiable and accessible biomarkers that could be used routinely.

Moreover, as many studies have demonstrated, the strength of a liquid biopsy is based on the association of several biomarkers (20). Thus, in the long term, this study and the discovery of various biomarkers such as plasma proteins, ctDNA, and blood cells would help clinicians choosing the best adapted treatment to each patient. More interestingly, the association of these biomarkers could provide a more reliable and powerful composite biomarker in TNBC.

## Author’s Note

The trial is managed by the Jean Perrin Centre, in Clermont-Ferrand, France.

## Ethics Statement

This study has been approved by the SUD-EST IV ethics committee and is conducted in accordance with the Declaration of Helsinki and General Data Protection Regulation (GDPR).

## Author Contributions

HV, XD, SL, YB, NR-R, IM, CA, and MB participated in the developing and conception of the study, and drafted the manuscript. XD is the coordinator of the study. XD and MB are medical leads. IM is the statistical lead, designed and will perform statistical analyses. HV is the project manager of the study and is involved in aspects of the day-to-day running of the trial. HV wrote the first draft of this manuscript. All authors contributed to the article and approved the submitted version.

## Conflict of Interest

The authors declare that the research was conducted in the absence of any commercial or financial relationships that could be construed as a potential conflict of interest.

## Abbreviations

ctDNA, circulating tumoral DNA; HER2, Human Epidermal Growth Factor Receptor 2; NAC, Neoadjuvant chemotherapy; NLR, Neutrophil-to-lymphocyte Ratio; PLR, Platelet-to-lymphocyte Ratio; TILs, Tumour infiltrating lymphocytes; TNBC, Triple negative breast cancer.

## References

[1] Lee J Kim D-M Lee A. Prognostic Role and Clinical Association of Tumor-Infiltrating Lymphocyte, Programmed Death Ligand-1 Expression With Neutrophil-Lymphocyte Ratio in Locally Advanced Triple-Negative Breast Cancer. Cancer Res Treat (2019) 51:649–63. 10.4143/crt.2018.270 PMC6473269 30064200

[2] Dent R Trudeau M Pritchard KI Hanna WM Kahn HK Sawka CA. Triple-Negative Breast Cancer: Clinical Features and Patterns of Recurrence. Clin Cancer Res (2007) 13:4429–34. 10.1158/1078-0432.CCR-06-3045 17671126

[3] Foulkes WD Smith IE Reis-Filho JS. Triple-Negative Breast Cancer. N Engl J Med (2010) 363:1938–48. 10.1056/NEJMra1001389 21067385

[4] Garrido-Castro AC Lin NU Polyak K. Insights Into Molecular Classifications of Triple-Negative Breast Cancer: Improving Patient Selection for Treatment. Cancer Discov (2019) 9:176–98. 10.1158/2159-8290.CD-18-1177 PMC6387871 30679171

[5] Loi S Drubay D Adams S Pruneri G Francis PA Lacroix-Triki M. Tumor-Infiltrating Lymphocytes and Prognosis: A Pooled Individual Patient Analysis of Early-Stage Triple-Negative Breast Cancers. J Clin Oncol (2019) 37:559–69. 10.1200/JCO.18.01010 PMC7010425 30650045

[6] Luen SJ Salgado R Dieci MV Vingiani A Curigliano G Gould RE. Prognostic Implications of Residual Disease Tumor-Infiltrating Lymphocytes and Residual Cancer Burden in Triple-Negative Breast Cancer Patients After Neoadjuvant Chemotherapy. Ann Oncol (2019) 30:236–42. 10.1093/annonc/mdy547 30590484

[7] Dieci MV Radosevic-Robin N Fineberg S van den Eynden G Ternes N Penault-Llorca F. Update on Tumor-Infiltrating Lymphocytes (Tils) in Breast Cancer, Including Recommendations to Assess TILs in Residual Disease After Neoadjuvant Therapy and in Carcinoma in Situ: A Report of the International Immuno-Oncology Biomarker Working Group on Breast Cancer. Semin Cancer Biol (2018) 52:16–25. 10.1016/j.semcancer.2017.10.003 29024776

[8] Salgado R Denkert C Demaria S Sirtaine N Klauschen F Pruneri G. The Evaluation of Tumor-Infiltrating Lymphocytes (Tils) in Breast Cancer: Recommendations by an International Tils Working Group 2014. Ann Oncol (2015) 26:259–71. 10.1093/annonc/mdu450 PMC6267863 25214542

[9] von Minckwitz G Untch M Blohmer J-U Costa SD Eidtmann H Fasching PA. Definition and Impact of Pathologic Complete Response on Prognosis After Neoadjuvant Chemotherapy in Various Intrinsic Breast Cancer Subtypes. J Clin Oncol (2012) 30:1796–804. 10.1200/JCO.2011.38.8595 22508812

[10] Ghajar CM. Metastasis Prevention by Targeting the Dormant Niche. Nat Rev Cancer (2015) 15:238–47. 10.1038/nrc3910 PMC4842412 25801619

[11] Redig AJ McAllister SS. Breast Cancer as a Systemic Disease: A View of Metastasis. J Intern Med (2013) 274:113–26. 10.1111/joim.12084 PMC3711134 23844915

[12] Benoy IH Salgado R Dam PV Geboers K Marck EV Scharpé S. Increased Serum Interleukin-8 in Patients With Early and Metastatic Breast Cancer Correlates With Early Dissemination and Survival. Clin Cancer Res (2004) 10:7157–62. 10.1158/1078-0432.CCR-04-0812 15534087

[13] Endo M Yamamoto Y Nakano M Masuda T Odagiri H Horiguchi H. Serum ANGPTL2 Levels Reflect Clinical Features of Breast Cancer Patients: Implications for the Pathogenesis of Breast Cancer Metastasis. Int J Biol Markers (2014) 29:239–45. 10.5301/jbm.5000080 24585434

[14] Noman AS Uddin M Chowdhury AA Nayeem MJ Raihan Z Rashid MI. Serum Sonic Hedgehog (SHH) and Interleukin-(IL-6) as Dual Prognostic Biomarkers in Progressive Metastatic Breast Cancer. Sci Rep (2017) 7. 10.1038/s41598-017-01268-4 PMC5431756 28496132

[15] Bahhnassy A Mohanad M Shaarawy S Ismail MF El-Bastawisy A Ashmawy AM. Transforming Growth Factor-β, Insulin-Like Growth Factor I/insulin-like Growth Factor I Receptor and Vascular Endothelial Growth Factor-a: Prognostic and Predictive Markers in Triple-Negative and non-Triple-Negative Breast Cancer. Mol Med Rep (2015) 12:851–64. 10.3892/mmr.2015.3560 PMC4438878 25824321

[16] Ethier J-L Desautels D Templeton A Shah PS Amir E. Prognostic Role of Neutrophil-to-Lymphocyte Ratio in Breast Cancer: A Systematic Review and Meta-Analysis. Breast Cancer Res BCR (2017) 19. 10.1186/s13058-016-0794-1 PMC5217326 28057046

[17] Huszno J Kołosza Z Mrochem-Kwarciak J Zajusz A. Prognostic Value of the Neutrophil-Lymphocyte, Platelet-Lymphocyte, and Monocyte-Lymphocyte Ratios in Male Breast Cancer Patients. Oncology (2020) 98:1–6. 10.1159/000505627 32344419

[18] Chae S Kang KM Kim HJ Kang E Park SY Kim JH. Neutrophil–Lymphocyte Ratio Predicts Response to Chemotherapy in Triple-Negative Breast Cancer. Curr Oncol (2018) 25:e113–9. 10.3747/co.25.3888 PMC5927790 29719435

[19] Patel DA Xi J Luo J Hassan B Thomas S Ma CX. Neutrophil-to-Lymphocyte Ratio as a Predictor of Survival in Patients With Triple-Negative Breast Cancer. Breast Cancer Res Treat (2019) 174:443–52. 10.1007/s10549-018-05106-7 30604000

[20] Alix-Panabières C Pantel K. Clinical Applications of Circulating Tumor Cells and Circulating Tumor DNA as Liquid Biopsy. Cancer Discov (2016) 6:479–91. 10.1158/2159-8290.CD-15-1483 26969689

[21] Beddowes E Sammut SJ Gao M Caldas C. Predicting Treatment Resistance and Relapse Through Circulating DNA. Breast (2017) 34:S31–5. 10.1016/j.breast.2017.06.024 28694015

[22] Fernandez-Garcia D Hills A Page K Hastings RK Toghill B Goddard KS. Plasma Cell-Free DNA (cfDNA) as a Predictive and Prognostic Marker in Patients With Metastatic Breast Cancer. Breast Cancer Res BCR (2019) 21. 10.1186/s13058-019-1235-8 PMC6924016 31856868

[23] Chia SK Bramwell VH Tu D Shepherd LE Jiang S Vickery T. A 50-Gene Intrinsic Subtype Classifier for Prognosis and Prediction of Benefit From Adjuvant Tamoxifen. Clin Cancer Res (2012) 18:4465–72. 10.1158/1078-0432.CCR-12-0286 PMC3743663 22711706

[24] Vieira AF Schmitt F. An Update on Breast Cancer Multigene Prognostic Tests—Emergent Clinical Biomarkers. Front Med (2018) 5:248. 10.3389/fmed.2018.00248 PMC6131478 30234119

[25] Wallden B Storhoff J Nielsen T Dowidar N Schaper C Ferree S. Development and Verification of the PAM50-based Prosigna Breast Cancer Gene Signature Assay. BMC Med Genomics (2015) 8:54. 10.1186/s12920-015-0129-6 26297356 PMC4546262

[26] Soliman H Shah V Srkalovic G Mahtani R Levine E Mavromatis B. MammaPrint Guides Treatment Decisions in Breast Cancer: Results of the IMPACt Trial. BMC Cancer (2020) 20:81. 10.1186/s12885-020-6534-z 32005181 PMC6995096

[27] Mittempergher L Delahaye LJ Witteveen AT Snel MH Mee S Chan BY. Performance Characteristics of the BluePrint^®^ Breast Cancer Diagnostic Test. Transl Oncol (2020) 13:100756. 10.1016/j.tranon.2020.100756 32208353 PMC7097521

